# Rewiring, forgetting and learning. Commentary: A critical period for experience-dependent remodeling of adult-born neuron connectivity

**DOI:** 10.3389/fnins.2015.00298

**Published:** 2015-08-24

**Authors:** Alonso Martínez-Canabal

**Affiliations:** Cell Biology Department, Faculty of Sciences, National Autonomous University of MexicoMexico city, Mexico

**Keywords:** neurogenesis, hippocampus and memory, rabies virus, forgetting, enrichment

Thirty years after the discovery that neurons are generated in the adult brain (Altman and Das, 1965), studies of the link between hippocampal neurogenesis and behavior revealed that exercise and environmental enrichment are highly effective at maximizing the proliferation and survival of adult-born neurons (Van Praag et al., 1999b; Bruel-Jungerman et al., 2005). Depending on the neurogenesis markers used, enriched animals exhibit a 50–200% increase in the number of new neurons compared with animals housed in standard home cages. Based on the premise that these new cells are important for cognition, several experiments show that exercise or environmental enrichment results in noticeable improvements in hippocampal-dependent task performance (Van Praag et al., 1999a; Van der Borght et al., 2007; Creer et al., 2010). Furthermore, the depletion of adult-born neurons with antimitotic drug treatment or focal radiation blocks the effect of environmental enrichment on memory (Bruel-Jungerman et al., 2005; Meshi et al., 2006). However, in nearly all of these studies, enrichment occurred before learning but not after learning. In fact, this experimental design is routinely used in the functional study of neurogenesis, not only with enrichment but also with other manipulations.

However, two *in vivo* (Feng et al., 2001; Akers et al., 2014) and one *in silico* (Weisz and Argibay, 2012) experiment show that when environmental enrichment occurs after learning, previously-acquired memories are forgotten. These three studies show that as a consequence of more cells added to the hippocampal circuit, memory retention suffers. This seemingly counterintuitive result can be explained by a detailed analysis of the morphological changes driven by running and enrichment and their effect on neuronal connectivity. Specifically, newly generated axon terminals can juxtapose with or displace previously established synapses (Akers et al., 2014), thus degrading pre-existing memory circuits and resulting in forgetting.

By taking advantage of the rabies virus to retrogradely infect monosynaptic neurons (Wickersham et al., 2007), a new study by Bergami et al. (2015) shows that this neurogenesis-induced rewiring occurs more extensively than previously thought. The authors created a retrovirus that tags both newly generated neurons and all neurons that have synaptic contacts with these new neurons with a TVA receptor, allowing tagged neurons to be labeled with permanently expressed GFP. They then counted the number of retroinfected cells within the dentate gyrus, the entire hippocampus, and other areas of the brain. They found that the number of cells that made synaptic contact with newborn hippocampal neurons was higher after environmental enrichment. Newly generated neurons in enriched mice received more innervation from cells in the dentate gyrus, as well as increased innervation from populations of inhibitory neurons in the hippocampus. Furthermore, newborn neurons in enriched mice received more innervation from the entorhinal cortex, mammillary bodies, and septal nuclei.

Previous studies show that newly formed synapses between adult-generated dentate gyrus neurons and CA3 pyramidal neurons can compete with or even displace previously-established synapses (See references in Deng et al., 2010). The mechanism by which new synapses juxtapose with or displace new synapses is unknown. However, assuming that the displaced synapses critically support a pre-existing memory circuit, such competition and displacement could explain why neurogenesis destabilizes previously acquired memories and leads to forgetting (Frankland et al., 2013; Akers et al., 2014). If we consider that forgetting can be adaptive in an ever-changing environment, we can conclude that neurogenesis is a process that actively supports the disruption of memory in response to environmental stimulation.

Bergami et al. (2015) show that new neurons generated by environmental enrichment are eager to form new synapses, not only within the canonical DG-CA3 circuit but also with more distant regions of the brain involved in cognition. Therefore, it is likely that this neurogenesis-induced restructuring of brain circuitry would not only induce forgetting of pre-existing memories but also enhance the formation of new memories. In a natural environment, therefore, the hippocampus may constantly be generating new memories while at the same time constantly erasing memories that are not exported and consolidated in cortical modules, as suggested by Kitamura and Inokuchi (2014). This constant rewiring is costly and complex and thus may only be active when there is a strong cognitive demand. Bergami et al. (2015) show that when such cognitive demand exists due to environmental enrichment, a hippocampal neurogenesis-induced rewiring program predominates and even modifies the way in which hippocampal neurons communicate with cells in other structures. Although there is a need for further studies to elucidate how hippocampal neurogenesis impacts distant brain areas, adult-generated neurons appear to coordinate a dynamic rewiring of memory circuits that include several forebrain areas.

## Conflict of interest statement

The author declares that the research was conducted in the absence of any commercial or financial relationships that could be construed as a potential conflict of interest.
